# Tailoring the Ablative Strategy for Atrial Fibrillation: A State-of-the-Art Review

**DOI:** 10.1155/2022/9295326

**Published:** 2022-02-28

**Authors:** Zefferino Palamà, Martina Nesti, Antonio Gianluca Robles, Antonio Scarà, Silvio Romano, Elena Cavarretta, Maria Penco, Pietro Delise, Mariano Rillo, Leonardo Calò, Luigi Sciarra

**Affiliations:** ^1^Electrophysiology Unit, Casa di Cura “Villa Verde”, Via Golfo di Taranto, 22, Taranto, Italy; ^2^Department of Life, Health and Environmental Sciences, University of L'Aquila, L'Aquila, Italy; ^3^Cardiovascular and Neurological Department, Ospedale San Donato, Via Nenni, 20/22, Arezzo, Italy; ^4^Cardiology Unit Ospedale “Di Venere”, Bari, Italy; ^5^Cardiology Unit, Policlinico Casilino, Via Casilina, Rome 1049, Italy; ^6^Department of Medico-Surgical Sciences and Biotechnologies, Sapienza University, Latina, Italy; ^7^Mediterranea Cardiocentro, Napoli, Italy; ^8^U.O. Cardiologia, Ospedale P. Pederzoli, Peschiera Del Garda (VR), Italy

## Abstract

In spite of technological progress and the improving skills of operators, atrial fibrillation (AF) ablation results appear to date to be at a plateau. In any case, the superiority of ablation over pharmacological therapy in terms of effectiveness, reduction of hospitalizations, and improvement has been well demonstrated in recent randomized trials. Triggers, substrate, and modulating factors (elements of Coumel's triangle) play different roles in paroxysmal and persistent AF, so induction and perpetuation mechanisms of arrhythmia may be different in each patient. Although effective ablative strategies are available for the treatment of paroxysmal AF triggers and persistent AF substrates, an adequate clinical evaluation of the patient is crucial in order to increase the chances of success. Recognizing triggers allows not only performing an effective ablation but also to avoid unnecessary lesions and at the same time reducing the risk of complications. AF beginning and triggers could be recorded by 12-lead ECG, continuous Holter monitoring, or implantable devices. In case of an unsuccessful noninvasive evaluation, nonpulmonary vein triggers should be investigated with an electrophysiological study. Persistent AF needs more effort to perform an accurate substrate characterization. Among the many methods proposed, recently the use of high-density mapping and multipolar catheters seems of particular benefit in order to clarify the arrhythmia mechanisms. Surgical and hybrid techniques allow to treat regions such as the posterior wall or Bachmann's bundle, which is fundamental for an ablative strategy that goes beyond just pulmonary vein isolation. Too often, patients are referred to electrophysiology laboratories without adequate preprocedural screening and planning in order to submit them to a standard “ready-made” procedure. The accurate search for triggers in paroxysmal AF and the correct recognition of the link between a possible underlying heart disease and the substrate in persistent AF could allow us to tailor the interventional approach in order to overcome the current plateau, increasing ablative procedure success and minimizing complications.

## 1. Introduction

Atrial fibrillation (AF) is the most common sustained arrhythmia, affecting approximately 3% of the adult population and nearly 6% of those over the age of 65 [1]. AF is associated with an increased risk of stroke, heart failure, reduced survival and quality of life [2]. Commonly, AF is classified on the basis of episode duration. Thus, AF can be classified as paroxysmal (lasting <7 days), persistent (lasting >7 days or requiring cardioversion at any time), long-standing persistent (uninterrupted AF for more than 1 year for which a rhythm control strategy is envisaged), and permanent (uninterrupted AF for which a rhythm control strategy is not envisaged) [3]. The pathophysiology of AF is complex and still not fully understood. In the 1960s, Coumel et al. described the mechanism of arrhythmias on the basis of the interaction of 3 elements: triggers, arrhythmogenic substrate, and modulating factors, which formed the so-called “Coumel's triangle” [4, 5]. These three elements play a different role in the pathogenesis of arrhythmias, and understanding the main mechanisms of each arrhythmia can help the physician select the best individual therapeutic approach [6]. Currently, each of these mechanisms can play a role in the AF scenario, such as the presence of triggers and a modified substrate for AF maintenance [7, 8]. Anyway, the correlation between AF temporal classification and pathophysiology is poor, even if more frequently the paroxysmal form is trigger-related and the persistent one is mainly considered to be substrate-based. Even extracardiac factors such as the autonomic nervous system (modulating factor) can play a role in the triggering of vagal atrial fibrillation. Some patients may have a prevalent vagal AF form and, in those cases, parasympathetic ganglia ablation could represent an effective ablation strategy as a modulating factor treatment [9]. Other AF forms can be related to a marked release of catecholamines that occurs as a result of both the intense physical effort and the emotional stress involved in the competition [10].

Over the past 20 years, catheter-based, surgical, and hybrid ablation techniques have been shown to be more effective than drug therapy in achieving rhythm control in patients with AF [11–13]. In the RAAFT study, which enrolled patients not previously treated for AF, the superiority of ablative treatment in terms of relapses has been demonstrated (patients on drug therapy 63% relapse and ablative therapy 13%) [14]. The CABANA study enrolled 2204 patients in 126 participating centers in 10 different countries chosen among experienced centers. Patients (median age 67 years) were randomly assigned 1 : 1 to ablation (*n* = 1108) or drug therapy (*n* = 1096); in the pharmacological therapy arm, it was possible to choose between rhythm and frequency control, according to the guidelines. Patients with all types of AF form were eligible, but persistent AF was the most prevalent form (47.3%), with an average time from the onset of arrhythmic history of >1 year, and only 10% of patients were completely asymptomatic. The median follow-up (FU) was approximately 4 years. No significant differences were found in the intention-to-treat (ITT) analysis for the primary composite endpoint (death, disabling stroke, major bleeding, or cardiac arrest) due to ablation against therapy pharmacological (8% vs. 9.2%, *p*=0.30) as well as for death (5.2% vs. 6.1%, *p*=0.38) or disabling stroke (0.3% vs. 0.6%, *p*=0.19). On the other hand, in the ITT analysis, the composite secondary endpoint (cardiovascular (CV) death or hospitalization) was reduced in the ablation arm (51.7% vs. 58.1%; *p*=0.001*P* = 0.001) and the time endpoint to the first AF recurrence was reduced by 48% (HR 0.52; *p* < 0.0001) [15]. In the EAST-AFNET4 study, patients were randomized in a 1 : 1 proportion to rhythm-control therapy (using an antiarrhythmic drug or ablation) or usual therapy (typically rate control therapy). At the cutoff (median 5.1 years), the first primary endpoint of a CV event—as CV death, stroke, or hospitalization for heart failure or ACS—occurred at a rate of 3.9 per 100 person-years in the rhythm-control group versus 5 per 100 person-years in the usual care group (HR 0.79; 95% CI: 0.66–0.94) [16].

However, despite technological progress and the improved skills of operators, too often patients have approached first-line ablative treatment without an adequate clinical evaluation or electrophysiological exploration. The effect of this procedural standardization without correct clinical screening and subsequent tailored planning is that postablation success rates appear to be at a “plateau,” particularly for long-term follow-up. Furthermore, the price we pay in terms of real-word procedural complications does not seem to be irrelevant [17].

Although it is possible to enact the ablative treatment of triggers in paroxysmal AF and that of the substrate in persistent AF, we should consider the clinical features of each patient in order to improve the interventional procedure outcome. Improvement of modifiable risk factors for AF is important in reducing AF recurrences; these include smoking, hyperlipidemia, hypertension, diabetes mellitus, sleep apnoea, obesity, excessive alcohol use, hyperthyroidism, pulmonary disease, air pollution, heart failure, and endurance exercise [3].

In order to reduce the complication rates and improve the efficacy/risk ratio, our group tried to propose different ablative approaches in different candidates to ablation according to the specific mechanisms of the arrhythmia in different patient subgroups. By tailoring the ablative strategy in the single clinical setting, it may be possible to overcome the plateau achieved in terms of the success of AF ablation and to significantly increase the benefits/risks ratio.

## 2. Paroxysmal AF

Paroxysmal AF is defined as lasting <7 days (generally 24–48 h) regardless of whether or not an intervention is needed for its resolution. The short duration of the arrhythmias reflects a self-limiting condition [3], and it is related to the fact that the main arrhythmia mechanisms are the presence of ectopic triggers with little or no abnormal atrial substrate. AF triggers are usually represented by ectopic atrial foci. However, any type of supraventricular tachycardia (e.g., atrial tachycardia-focal or reentry, atrial flutter, intranodal reentry, and atrioventricular bypass-mediated tachycardias) can play a triggering role for AF [18]. Patients with atrial ectopic triggers inducing AF were the subjects of the work by Haissaguerre et al. published in 1998 that opened the pulmonary vein isolation (PVI) era as the cornerstone for AF ablation techniques [6]. In 45 patients with frequent episodes of purely paroxysmal AF and frequent atrial ectopic beats, the origin of the ectopic beats was found to be mostly confined to the pulmonary veins (in 95% of cases), with the origin of the ectopic beats in the pulmonary vein sleeves and the ablation of these ectopic foci resulting in the absence of recurrent AF in 62% of cases after 7 months of follow-up [6].

The left atrium walls are made up of 1 to 3 muscle layers that extend up to 2 cm inside the pulmonary vein ostia (PVs) [19]. At the level of these venous-atrial connections, there are some muscular sleeves that constitute an arrhythmogenic substrate as the anatomical, histological, and electrical discontinuities (between PV and left atrium) favor microreentry mechanisms at the basis of rapid focal ectopic triggered activity. On 12-lead ECG, this early ectopic activity is recognizable with the so-called “P on T” phenomenon, with very early extra systolic atrial beats [20]. Ectopic triggers do not originate only from PVs, but less frequently from the right atrium, superior vena cava, interatrial septum, coronary sinus, and the Marshall vein [21, 22].

**Figure 1:**
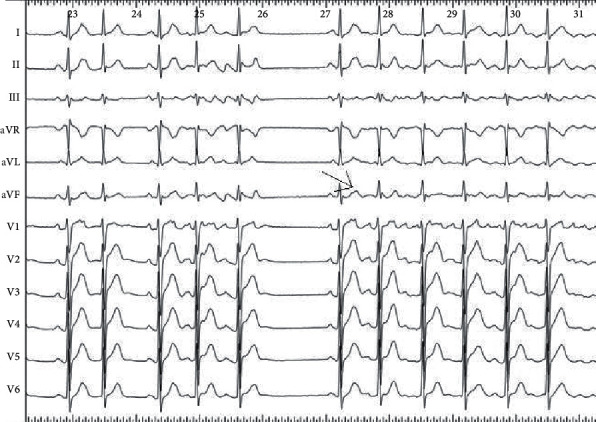
“P on T” phenomena: the trigger of arrhythmia is an extra systolic nonconducted atrial beat with a negative P in D1 and aVL and a positive in D2, D3, and aVF (arrow). This suggests an origin from the left (negative P in D1 and aVL) superior (positive in D2, D3, and aVF) pulmonary vein that was confirmed by the Lasso catheter. The isolation of the left superior pulmonary vein stopped the arrhythmia.

**Figure 2:**
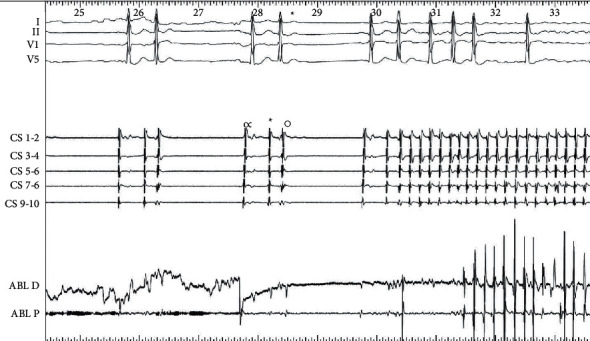
The trace shows the trigger of atrial fibrillation. It seems due to an extra systolic beat (^*∗*^), but the endocardial electrogram shows the following sequence: sinus beat (*∞*), extra systolic beat (^*∗*^), and blocked extra systolic beat (°). This latter is the effective trigger of atrial fibrillation (CS: coronary sinus; ABL: ablator; D: distal; P: proximal).

The analysis of triggers origin is important not only to perform an effective ablation for paroxysmal AF but also to avoid unuseful and potentially harmful lesions and reduce related complications [6]. Sometimes an accurate analysis of the surface ECGs, including capturing of the AF beginning, can reveal the trigger (Figures 1 and 2). Otherwise, the first step is an accurate patient interview. Anamnesis is the lowest cost procedure in medicine and probably the most cost-effective. It could facilitate the discovery that AF (arrhythmic palpitations) is preceded by regular palpitations or that the arrhythmia is unleashed by a physical effort, or after a big meal, or at night while sleeping, and so on.

Furthermore, it is necessary to consider all the available tools that can help to identify the mechanisms that trigger or support AF. Invasive and noninvasive ECG monitoring systems, involving continuous Holter monitoring, external event and/or loop recorders, and implantable devices can significantly help to study AF triggers or arrhythmia mechanisms in general. When these tests are not diagnostic, it may be reasonable to perform an electrophysiological study before performing PVI in order to investigate nonpulmonary vein triggers.

However, there are possible pitfalls to the tailored approach. The most important thing is that it can be time-consuming and therefore can delay the ablative strategy. However, it should be considered that only a minority of patients with paroxysmal AF evolve towards persistent forms, and this is particularly true in young patients with “lone AF” [23].

**Figure 3:**
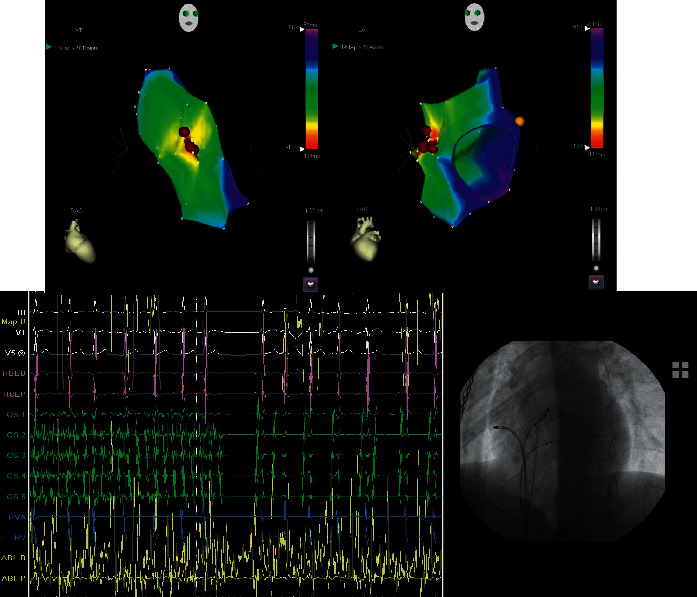
In the upper panels, Carto V2 (Biosense Webster, Johnson and Johnson) activation maps show the maximum earliness (red zone) of the origin of tachycardia in the right atrium (crista terminalis). In the lower panel, traces during radiofrequency delivery (during AF) with the restoration of sinus rhythm and the appearance of irritative beats identical to tachycardia. In the lower right panel, fluoroscopy during radiofrequency delivery.

In any case, once the trigger is recognized in a precise and reproducible manner, its treatment is able to control AF recurrences, guaranteeing a good outcome in terms of AF free survival, and this kind of approach is always superior to any “empirical” ablative treatment [24]. In fact, the lone triggering-arrhythmia ablation has been shown to be effective in preventing AF recurrence with success rates >90% even in long follow-up studies [25]. In Figure 3, it is represented by an atrial tachycardia from the right atrium (crista terminalis) capable of triggering episodes of paroxysmal AF. The delivery of radiofrequency during fibrillation at the site of trigger origin also stops the fibrillation.

Although the inability to find a precise trigger is a limitation of the tailored ablative approach, a reasoned analysis of arrhythmia would always be desirable to screen which patients could benefit from a tailored approach rather than an empirical PVI.

## 3. Persistent AF

The results of persistent and long-standing persistent AF ablation are currently unsatisfactory, especially for especially the latter, and success rates vary between 50% and 60% for persistent AF [26]. Although PVI remains a cornerstone of any AF ablation [8, 27, 28], other regions of the left atrium, i.e., non-PV substrates, are involved in AF, particularly in persistent AF [29]. In contrast with the paroxysmal AF, where the role of the trigger is prevalent, the persistent AF is mainly and decisively influenced by another factor of the Coumel triangle [5]: the substrate. However, substrate modification that significantly impacts the outcome of persistent AF patients has not been documented in previously published studies [29].

### 3.1. Targeting Persistent AF Mechanisms

Complex fractionated atrial electrogram (CFAE), rotors ablation, and additional linear lesion did not clearly demonstrate benefit when compared to PVI only (STAR-AF2) [30]. One limitation of substrate modification is that it is always difficult to categorize and recognize patients who can benefit from this approach. Some clinical settings, such as patients with “diabetic heart disease,” show the presence of a more important substrate component like CFAEs [31]. Other settings show an evolution of the substrate related to the progression of the disease, as in the case of rheumatic heart disease and consequent rheumatic atrial myopathy [32, 33]. However, all studies have considered too small sample sizes to define a standard of care in every particular scenario. Therefore, it appears difficult to propose a standardized and, above all, reproducible approach to persistent AF ablation. In recent years, however, technological evolution has been directed not only towards ablative techniques with different sources of energy, but also in the ability to map more and more precisely substrates located in the left and right atrium. High-density endocardial voltage mapping using multipolar catheters has been increasingly used in clinical practice to identify left atrial anatomical areas of low-voltage electrical activity [34, 35], which is commonly considered a marker of atrial fibrosis [36]. This allows us to see with different eyes, with a magnifying glass, what we could not see in the past years. Left atrial substrate modification by targeting low-voltage zones is an ablation strategy that, in addition to PVI, tries to erase arrhythmogenic mechanisms harbored in such tissue [34, 36–38]. The common feeling is that PVI alone is not sufficient; therefore, some of the new techniques could allow us to better target a tailor-made ablative treatment, adapting the ablation to the characteristics of the different patients and their substrates. In several recent studies, left atrial bipolar endocardial voltage maps constructed by acquiring thousands of voltage points [39] have emerged as a promising tool for defining AF substrates during radiofrequency-ablation procedures [40]. Indeed, the low-voltage zone has been considered as a surrogate marker of the presence of atrial fibrosis and may play a role in giving rise to the mechanisms underlying AF, especially in the case of persistent AF [41, 42]. In our view, this approach offers a simple method to look for potential and selective electroanatomical substrates underlying AF and probably constitutes the basis of truly tailored ablation in persistent AF patients. Indeed, this approach, which was both qualitative and quantitative, could help us to identify patchy fibrosis, which could represent local non-PV conductive alterations that influence anisotropy during atrial fibrillation. Moreover, in order to perform an accurately tailored substrate ablation, we cannot ignore the correct identification of the mechanisms underlying the arrhythmia such as rotational activity. In the AF rotor, the primary mechanism is its core that activates in small areas with emanating spiral arms that disorganize and fuse with the surrounding activation. Focal impulse and rotor modulation (FIRM) mapping represents an ablation strategy based on recognition and subsequent removal of rotors, along with subsequent FIRM mapping, in order to confirm their elimination [43]. Initial attempts by investigators to use isochronal (activation) mapping did not detect stable rotational circuits in the past experience [44], but to date, new multipolar catheters and software allowed us to understand the AF mechanism, identifying the rotors more and more precisely [45, 46]. Although there is no current standardization of the treatment of patients with persistent AF and without prejudice to the PVI cornerstone, a reasoned approach guided by high-density mappings can allow tailoring the ablative strategy to the individual patient. Although the substrate modification has given the feeling of having a role in the treatment of persistent and long-standing persistent AF, to date, there is insufficient scientific evidence to propose this ablative treatment without PVI. New technologies are supporting an increasingly fine substrate characterization both in terms of careful recognition of areas of fibrosis and recognition of the AF substrate. However, the complex interaction between any underlying heart disease and the substrate is difficult to classify, which is why the treatment of persistent AF is still too often empirical but should be better tailored to the individual patient.

**Figure 4:**
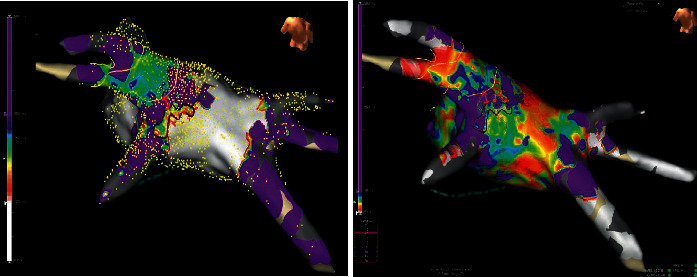
High-density mapping (12,275 points) performed with the 3D Precision System and HDGrid omnipolar catheter (Abbott) to research during atrial fibrillation of rotational activity (RA). The figure on the left shows a map during AF that allows identifying areas of interest compatible with rotational activity (cycle length mean map, CLmean), with activity between 120 and 300 msec. Areas of interest appear in sets of different colors, while insignificant areas appear as white and purple (below 120 msec and above 300 msec, respectively). On the right side, the figure shows the standard deviation map (SD map) of the same activity during AF (cycles length between 0 and 50 msec), in order to establish whether the rotational activity identified with the CLmean (at the level of the posterior wall near the left inferior pulmonary vein) is stable over time. The set of different colors indicates stable rotational activity in this region (SD between 0 and 50 msec).

**Figure 5:**
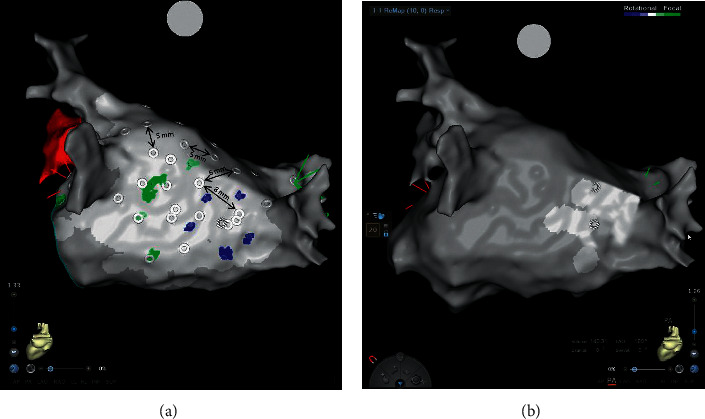
(a) High-density mapping performed with the 3D CARTO 3 V7 system and bipolar PentaRay™ NAV Catheter (Biosense Webster), to research, during AF, rotational (Ras), and focal activities (FA). The green squares indicate the FAs, while the blue squares indicate the RAs. A repetitive RA is clearly visible between the posterior and the inferior wall of the left atrium, involving a large area (calculated at 4.2 cm^2^), which is more compatible with a real rotor (macroreentrant driver rather than a microreentrant one). Positioning the PentaRay in the same spot at different times (white circles with a distance of ≤1 mm), we reproduced the left atrium identically. The ablation was directed to the treatment of the RAs. In (b), after RF application on the entire rotor seat area, the left atrium mapping during fibrillation demonstrates its complete disappearance (FIRM approach). The procedure was completed with PVI and synchronized electrical cardioversion. No AF recurrence at 12-month follow-up.

We report two persistent AF patients with left atrium dilation, the first using the 3D NavX Precision system + the HDGrid catheter for mapping, and the second using the 3D CARTO 3 V7 system + the PentaRay catheter. In both cases, attention was paid to mechanisms that can allow atrial fibrillation persistence. In fact, over the last decade, the field has shifted focus to panoramic mapping techniques that have identified 2 arrhythmic mechanisms of interest, namely, rotational activity (RA, rotors) and ectopic focal activations (FA) as drivers of AF (Figures 4 and 5).

### 3.2. Anatomical Persistent AF Ablation

Beyond PVI and attempts to understand and to treat the persistent AF mechanisms, other atrial anatomical structures can be considered targets, either by catheter ablations, by surgical techniques, or better yet, with hybrid approaches. If, from a purely conceptual point of view, the reduction of the atrial critical mass operated by extensive ablations should improve the outcome in terms of AF free survival, the results of the STAR-AF-2 reaffirmed the concept “less may be more” [30]. However, to date, transcatheter ablative approaches with mitral, roof, and cavotricuspid isthmus block have been proposed with the addition of coronary sinus and vein of Marshall (CS-VOM) musculature treatment (even with ethanol infusion) [47].

The posterior wall also plays a critical role in the initiation and maintenance of AF. In fact, its myocardial extensions into the PVs share similar embryologic origins and specialized conduction tissue derived from the heart tube with intrinsic pacemaker activity [48]. The transcatheter approach to posterior wall isolation, however, in addition to being technically more complex to achieve, is burdened by a high-risk of complications due to its proximity to structures such as the esophagus and periesophageal branches of the vagus nerve. Furthermore, an empirical transcatheter complete posterior wall isolation does not improve the rhythm outcome of catheter ablation or influence the type of recurrent atrial arrhythmia (probably linked to an incomplete isolation of the wall itself) [49].

However, surgical, or better, hybrid ablations (in a single session or staged) have been shown to maximize the collaboration between electrophysiologists and surgeons in the AF treatment. Some structures such as the posterior wall can in fact be approached by the epicardium in an effective way (ensuring transmurality and homogeneity of the lesions) and a safe way (by delivering energy from the outside to the inside without affecting structures such as the esophagus) using technologies such as the convergent procedure [50].

Recently a single-stage endo/epicardial ablation (the Mediterranea approach) has been proposed in order to target multiple endocardial as well as epicardial components closely related to AF sustaining (Bachmann's bundle and ligament of Marshall) [51].

## 4. Conclusion

Atrial fibrillation is a complex and multifactorial arrhythmia, which is different between various patients. Despite the several technological advances and the ever increasing experience of the operators, the AF ablation outcomes seem to be at a plateau. Although the complication rate is low, this rate remained unchanged over time. Furthermore, even if rare, some complications are hardly acceptable, as recently demonstrated in the ESS–PRAFA snapshot [52]. On the one hand, numerous technological and organizational efforts have been made in order to improve the quality and performance of electrophysiology laboratories [53], but few efforts have been made to characterize every single patient before undergoing ablation with a thorough clinical and electrophysiological customized study. It is therefore important to take time to study the single patient in order to tailor an ablative strategy improving success rates and reducing complications. It is well known that the concept “atrial fibrillation begets atrial fibrillation” [54] is a delayed ablation strategy in order to better study the arrhythmia mechanism that could favor the progression to persistent or long persistent forms, but it has been demonstrated that only a minority of paroxysmal AFs evolve towards persistent forms and this is particularly true in young patients with “lone AF.” The progression rate can be very different in different patients, and it is not related to the initial temporal arrhythmia classification. Sometimes AF presents itself as a persistent form, sometimes it tends to keep a paroxysmal behavior, and in any case, this behavior is strongly influenced by the comorbidities (i.e., hypertension, diabetes, and obesity) [55]. Tailoring the AF ablative strategy is not always feasible, but all preprocedural efforts should always be made to assess the patient. It is therefore necessary to consider all the available tools that can help to identify the mechanisms that trigger or sustain AF, and when we do not have enough clinical information from the noninvasive tests, an electrophysiological study before ablation could be indicated, in order to investigate nonpulmonary vein triggers that allow to achieve long-term success rates far higher than a lone PVI ablation and also performing an often technically simpler procedure with lower complication rates.

Persistent AF ablation cannot be separated from a correct clinical framework in common with paroxysmal AF; however, it requires an additional effort during the treatment of the arrhythmia. A correct characterization of the substrate and, above all, of the mechanisms that sustain the arrhythmia is fundamental to program an ablative strategy that goes beyond PVI. In this view, technological evolution allows us to look at the substrate with new eyes: the new multipolar catheters and high-density mapping techniques. More purely anatomical ablations can also be proposed with surgical or better hybrid procedures in order to effectively and safely treat structures that are difficult to treat with totally endocardial procedures (posterior wall, Masrhall's ligament, or Bachmann's bundle).

Even the third actor of Coumel's triangle, modulating factors, should not be ignored, reserving the possibility of modifying the influence of the autonomous nervous system in patients with vagally-mediated AF (for example, at night or after large meals) by ablating the intramural ganglionated plexuses in addition to the standard ablative treatment [9–56].

Can careful research into the mechanisms of atrial fibrillation change the thromboembolic risk prevention strategy? Clinical guidelines for the management of AF state that the procedure should not be performed to obviate the use of oral anticoagulants [3]. In the analysis by Noseworthy et al., which included patients registered in a large administrative database of requests who underwent AF ablation between 2005 and 2014, the rate of oral anticoagulation therapy (OAT) discontinuation was higher among patients at low risk for stroke (CHA2DS2-Vasc score: 0 or 1), but the rate of discontinuation was also high in subjects with higher CHA2DS2-Vasc scores. While all patients were at increased risk of stroke/TIA/systemic embolism in the first 3 months if they discontinued OAT, only those with a CHA2DS2-Vasc score> 2 were at increased risk when they discontinued OAT beyond 90 days. These high-risk patients had an increased risk of stroke/TIA/systemic embolism when they stopped OAT after 3 months by 2.5 times compared to those who did not stop treatment (HR: 2.48; *P* < 0.05) [57]. However, it is possible to use a tailored approach to anticoagulation in those patients in whom the clear ablation of the trigger (such as atrial tachycardia and the Kent bundle) has proved effective, and a suspension of anticoagulation therapy could be hypothesized. This strategy will obviously be supported by further studies.

**Figure 6:**
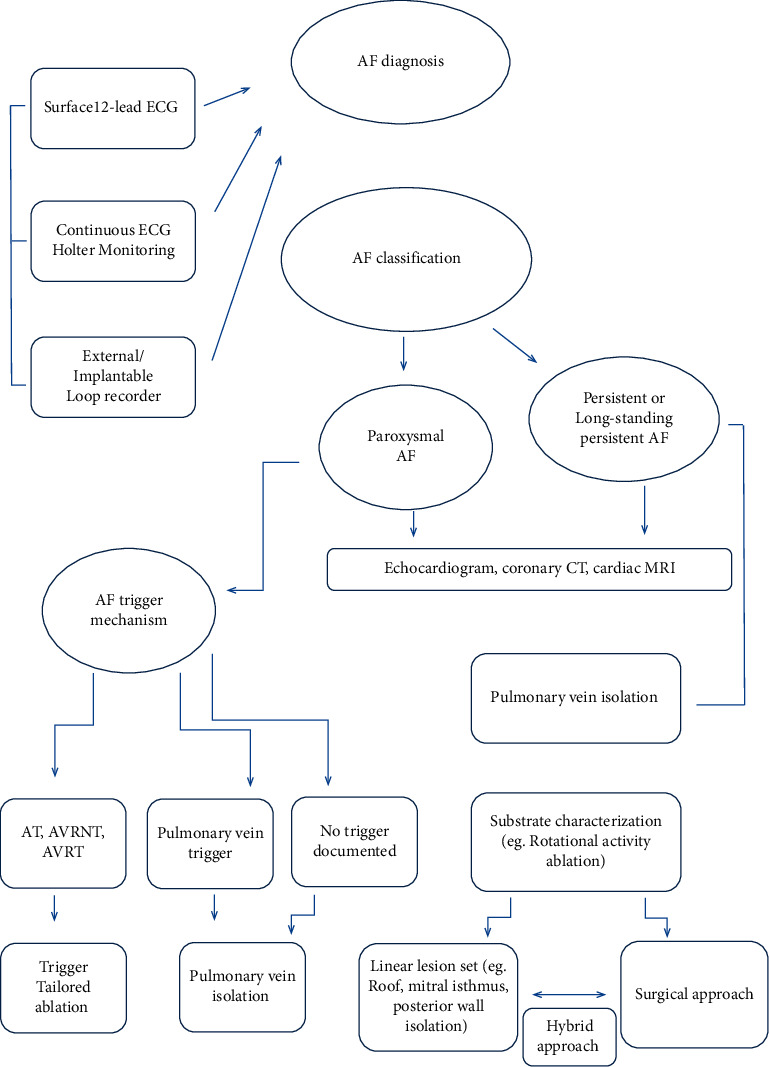
Stepwise approach to atrial fibrillation patient (AF: atrial fibrillation, AT: atrial tachycardia, AVRNT: atrioventricular reentrant nodal tachycardia, AVRT: atrioventricular reentrant tachycardia, CT: computed tomography, and MRI: magnetic resonance imaging).

Too often, the common practice of electrophysiology laboratories involves the direct referral of the patient to the interventional procedure without adequate preprocedural screening and planning in order to submit them to a standard “ready-made” procedure. A stepwise approach to the patient affected by AF should be proposed (Figure 6): the accurate search for triggers in paroxysmal AF and the correct recognition of the link between a possible underlying heart disease and the substrate in persistent atrial fibrillation could allow us to tailor the interventional approach in order to overcome the current plateau in terms of ablative procedure success and to minimize complications.

## Data Availability

All data are available from “cdc Villa Verde” Taranto and “Policlinico Casilino” Rome EP labs.
